# Modulatory effect of the exudates released by the brown kelp *Lessonia spicata* on the toxicity of copper in early developmental stages of ecologically related organisms

**DOI:** 10.1007/s11356-016-8120-0

**Published:** 2016-11-30

**Authors:** Alexandre Fellous, Santiago Andrade, Francisco Vidal-Ramirez, Ricardo Calderón, Jessica Beltran, Juan A. Correa

**Affiliations:** 1 Departamento de Ecología, Facultad de Ciencias Biológicas, Pontificia Universidad Católica de Chile, Av. Lib. B. O’Higgins 340, Santiago, Chile; 2 Université Pierre et Marie Curie (Sorbonne-Universités, Paris VI), 4 Place Jussieu, 75005 Paris, France; 3 Center of Applied Ecology and Sustainability (CAPES), Facultad de Ciencias Biológicas, Pontificia Universidad Católica de Chile, Av. Lib. B. O’Higgins 340, Santiago, Chile; 4 Estación Costera de Investigaciones Marinas La Cruces, Pontificia Universidad Católica de Chile, Osvaldo Marín 1672, Las Cruces, Comuna El Tabo, Región de Valparaíso, Chile; 5 School of Biological Sciences and Australian Research Council Centre for Excellence in Coral Reef Studies, The University of Queensland, St. Lucia, QLD 4072 Australia

**Keywords:** Kelps, Exudates, Copper, Crabs, Larvae, Spores

## Abstract

*Lessonia spicata* is a key dominant species along the Pacific coast of South America, providing a habitat for many organisms. However, this role can be affected by abiotic stress, such as metals. To counteract the toxic effect, *L. spicata*, among other seaweeds, releases exudates that bind metals. In this study, tolerances to copper of organisms related to the kelp forest (spores of *Ulva lactuca* (Chlorophyceae) and *L. spicata* (Phaeophyceae) and Zoea I of *Taliepus dentatus* (Milne-Edwards, Crustacea)) were studied; then, exudates are assessed by their protective effect. Exudates increase the 48-h 50% effective concentration (EC_50_) of the germination of spores from 8 to 23 μg Cu L^−1^ for *U. lactuca* and from 119 to 213 μg Cu L^−1^ for *L. spicata* and the survival of the larvae Zoea I 48-h 50% of lethal concentration (LC_50_) from 144 to 249 μg Cu L^−1^. Results indicated that exudates had a protective effect. Each species is specifically sensitive to copper. Crab larvae Zoea I were able to support higher doses, and exposure before hatching increased their tolerance.

## Introduction

A number of metals are considered essential micronutrients for organisms in marine environments. However, at elevated concentrations, metals become toxic (Stauber and Florence 1987; Festa and Thiele 2011). Copper is one of the best studied metals, and one of the first elements considered in the process of complexation (Donat and Van den Berg 1992; Wells et al. 1998). Dissolved copper in seawater occurs in a variety of physical and chemical forms, including free hydrated ions and various soluble inorganic and organic complexes of the metal, along with potential colloidal forms. However, copper ions occur complexed with organic ligands in a percentage higher than 99% in all aquatic environments (Coale and Bruland 1988). This fact is important because copper complexation, by organic ligands, decreases the levels of free copper ions reducing in consequence the availability and the potential toxicity of copper to marine organisms (Stauber and Florence 1987; Gledhill et al. 1997). Copper complexing ligands are components of the marine pool of dissolved organic matter (DOM). A few is known about the chemical structure or functional groups of organic copper complexing ligands in seawater, and the improvement in knowledge of this phenomena is crucial (Lohan et al. 2015). It is known that phytoplankton and macroalgae have the capacity to produce and release organic ligands to the surrounding seawater (Vasconcelos and Leal 2008; Karavoltsos et al. 2013). In the case of macroalgae, a relatively limited number of species have been studied and classified as potential sources of organic ligands, which are capable of complexing dissolved metals (Vasconcelos and Leal 2008; Andrade et al. 2010; Karavoltsos et al. 2013). In addition, some species such as *Chaetomorpha firma* (Chorophyceae), *Gelidium lingulatum* (Rodophyceae), and the brown kelp *Lessonia spicata* (Phaeophyceae) have the capacity to release exudates complexing copper with conditional stability constants (log k’) varied from 7.6 to 8.9 (weak ligands), both spontaneously and under metal stress (Andrade et al. 2010). Despite the fact that it is widely known that these compounds can decrease the toxicity of copper on marine organisms, this effect has not yet been quantified.

Copper is toxic to organisms because it may replace other metals in the metal-binding sites, interfering with correct functioning of proteins, and may trigger the production of reactive oxygen species (ROS) (Contreras et al. 2009a) affecting the growth and reproduction of marine organisms (Contreras et al. 2007; Paganni and Bianchini 2009). Also, it can interfere with the biosynthesis of the photosynthetic machinery, changing the protein and pigment composition of photosynthetic membranes (Aggarwal and Sharma 2011). It is known that the early developmental stages of organisms (larvae of invertebrates and fishes and the gametophytic phase of seaweeds) are considered the most susceptible to be affected by copper (Contreras et al. 2007; Paganni and Bianchini 2009).

Copper concentrations in marine environment are usually low. However, various sources of the metal, mostly due to anthropogenic inputs (industrial and domestic wastes, agricultural processes and discharges of mine activities), contribute to a progressive increase of copper in the seawater, especially in coastal ecosystems (Burridge and Bidwell 2002). For example, in the pacific coast of South America, northern of Chile, there is an area called the Chañaral Bay, where more than 150 t of copper mine tailings have been chronically discharged (Castilla 1996; Correa et al. 1999) and led to abnormally high dissolved Cu concentrations persisting in the environment (Andrade et al. 2006). It is also causing the disappearance of the brown kelp *Lessonia nigrescens*, which is considered as a ‘bio-engineer’ (Jones et al. 1994), diminishing the biodiversity on more than 20 km of coastline (Correa et al. 1999; Medina et al. 2005).

Recent molecular studies (Tellier et al. 2011; González et al. 2012) revealed that *L. nigrescens* is a complex that includes two cryptic species, the *L. berteroana* and *L. spicata*, of different distributions (16°S–30°S for *L. berteroana* and 29°S–42°S for *L. spicata*). These species form extensive kelp beds that have significant ecological importance (Steneck et al. 2002), which provides habitat for many seaweeds, invertebrates, and fishes in the intertidal zones of wave-exposed rocky shores along the Pacific coast of South America (Santelices 1980; Vasquez and Santelices 1984). Therefore, the negative effects of copper excess on these algae, despite their capacity to release exudates (Andrade et al. 2010), can directly or indirectly affect the entire coastal benthic community by the loss of organisms associated to the kelp declining biological diversity (Correa et al. 1999; Medina et al. 2005).

Among the large variety of invertebrates found in fronds and holdfast, the crab, called *Taliepus dentatus* (Crustacea), uses this seaweed as an exclusive habitat (Storch et al. 2009a). This is a common species dwelling the rocky shore habitats along the Peruvian and Chilean coasts (Storch et al. 2009b), which is mainly herbivorous (Pardo et al. 2007). The brown kelp serves as a source of food, refuge for oviparous females and their larvae, and also provides protection against desiccation, waves, and predation. In this context, it is possible to hypothesize that all species that live under the frond canopy of kelps, such as spores, microscopic gametophyte, and sporophytes of algae and larval stages of invertebrates, are protected.

In order to quantify the protective effect of *L. spicata* exudates on spores of the same species and in early developmental stages of ecologically related organisms, such as spores of the green alga *Ulva lactuca* and the larvae (Zoea I) of the crab *T. dentatus*, toxicological bioassays with copper, with and without exudates of *L. spicata*, were carried out. In addition, sensitivities of larvae from ovigerous females of *T. dentatus* exposed to copper before hatching were also explored.

## Material and methods

### Seawater preparation

Seawater was pumped from the shore at the *Estación Costera de Investigaciones Marinas* (ECIM) of Las Cruces (33°29′S and 71°38′W) using a submersible pump with plastic pipes passing through a system with successive filters (20, 10, 5, and 1 μm). Then, the seawater was filtered through a 0.45-μm polycarbonate membrane using an isolated filtration pump to prevent contamination. The first 1-L volume was used for cleaning the material followed by 40 L stored in the dark for 24 h at 15 °C for the culture media. After that, activated carbon was added in the ratio of 5 g per 20 L to remove the dissolved organic matter. After 24 h, seawater was filtered through 0.22-μm polycarbonate membranes and stored in the dark at 15 °C and used within a week. All materials were acid-cleaned (24 h in a detergent bath, followed by rinsing with distilled water, then 24 h in acid bath and dried under a hood) and stored in acid-cleaned plastic bags until use.

### *L. spicata* and *U. lactuca* assays

Algae were collected manually during low tide from the rocky shore at ECIM marine station.

#### Spore release of *L. spicata*

Individual disks were excised from selected *sori* (reproductive structures of sporophytes which release haploid spore that germinate into male- and female-independent gametophyte) obtained from mature fronds of *L. spicata* (Phaeophyceae). Each disk was washed three times with 0.22-μm filtered seawater. Then, they were dried with absorbent paper and air-dried at room temperature for 1 h to stimulate normal dissecation during low tide. They were placed in an acid-washed Erlenmeyer flask (250 mL) containing 200 mL of fresh seawater for the preparation of separated spore suspensions. When spore release occurred, disks were removed and cell density was measured using a hemocytometer (Neubauer chamber Boeco).

#### Spore release of *U. lactuca*

Individuals were washed three times with 0.22-μm filtered seawater and dried for 2 h at room temperature. To induce sporulation, each plant was placed in 2-L flasks with filtered seawater (0.22 μm) and positioned near the light source to concentrate the spores. Then, spores were collected and added to one side of an acid-washed Erlenmeyer flask with light to the other side. After 5 min, as a result of phototactic responses, spores that reached the other side were collected. This process removed other unicellular organisms which may be associated with the algal spores. Cell density was measured using a hemocytometer (Neubauer Chamber Boeco).

#### Effect of copper on spore germination of *U. lactuca* and *L. spicata*

For the bioassays of copper effects on spore germination of both macroalgae species, the same batch of spores was used. To perform the bioassay, a density of 50,000 spores mL^−1^ was inoculated into test chambers (25-mL Petri dishes, Pyrex), previously washed and containing 10 mL of test solution consisting of seawater with increasing copper concentrations, which prepared from dilutions of a standard solution of CuCl_2_ (Titrisol®, Merck).

Based on preliminary experiments, copper treatments were selected for each bioassay. A treatment of seawater with no added copper was also included as control. Each treatment was replicated three times. All cultures were maintained at 15 ± 1 °C, pH 7.6, 12 h light/12 h dark photoperiod, and an intensity of 40 ± 5 μmol photons m^−2^ s^−1^ under day-white fluorescent lighting (Philips TLT 20 W/54RS).

#### Enrichment of seawater with exudates of *L. spicata*

Juveniles of *L. spicata* (Phaeophyceae) were removed manually during low tide from the shore at ECIM marine station, using a plastic spatula to prevent mechanical damage. Samples were placed in acid-cleaned plastic bags with seawater from the site to minimize physiological stress and transported to the laboratory at 4 °C. Upon arrival at the laboratory, epiphytes and sediments particles were removed from the surface of the fronds by washing three times with 0.22-μm filtered seawater. Individuals of *L. spicata* were selected and placed in 2-L flasks with filtered seawater (0.22 μm). Therefore, algae were placed into the darkness for a night at 11 °C in order to acclimatize them to culture conditions. After that, seawater was replaced and algae were exposed for 48 h of treatment with continuous light at 11 °C, which is long enough for the releasing of exudates and adequate enough to avoid degradation of the tissues (Vasconcelos and Leal, 2008). The algal cultures used for exudate extraction were not enriched with nutrients. After 48 h, juveniles were removed, and the seawater enriched in exudates was filtered through 0.22-μm membranes, stored in the dark at 15 °C, and used within a week. Two flasks without algae were used as control. Light was used to maximize the natural production of exudates. A Teflon® film covered each flask and ventilation was done using an acid-cleaned pipette connected to air pump.

The production of exudates was tested through the determination of dissolved organic carbon (DOC) in the filtered seawater using a carbon analyzer (TOC Apollo 9000, Tekmar). The mean amount of DOC was 4.09 (±0.13) and 9.81 (±0.74) mg C L^−1^ in the filtered seawater with and without exudates, respectively.

#### The effect of *L. spicata* exudates on toxicity of copper on *U. lactuca* spore germination

First, 15 treatments consisting of filtered seawater with increasing copper concentrations and 1 treatment with no copper additions were included. Based on preliminary experiments, copper treatments included the nominal concentrations of 2, 3, 4, 6, 8, 10, 12, 15, 20, 24, 48, 80, 160, 300, and 500 μg Cu L^−1^ as CuCl_2_ (Titrisol®, Merck, Germany). Then, the same treatments consisting of filtered seawater enriched in *L. spicata* exudates with increasing copper concentrations and a treatment with no added copper were included to test the protective effect of the exudates.

#### The effect of *L. spicata* exudates on toxicity of copper on *L. spicata* spore germination

First, 12 treatments consisting of filtered seawater with increasing copper concentrations and 1 treatment with no copper additions were included. Based on preliminary experiments, copper treatments included the nominal concentrations of 15, 25, 43, 50, 76, 100, 120, 200, 300, 500, 700, and 1000 μg Cu L^−1^ as CuCl_2_ (Titrisol®, Merck, Germany). Then, the same treatments consisting of filtered seawater enriched in *L. spicata* exudates with increasing copper concentrations and a treatment with no added copper were included to test the protective effect of the exudates.

### *T. dentatus* assay

#### Female collection and spawning

Ovigerous females of *T. dentatus* (Milnes-Edwards, Crustacea) were collected by SCUBA diving, between 3 and 6 m depth at *Punta de Tralca* (33°25′8″S and 71°41′44″W), central Chile. The animals were brought to the laboratory at the ECIM marine station, where all experiments were conducted. Females were held in 50-L containers with running seawater at 15 °C, constant aeration and 12 h light/12 h dark photoperiod until the egg maturation. Then, after checking the maturity of the eggs, mature females were placed into 20-L containers using the same parameters until spawning and larval collection. The acclimation temperature was selected using the mean annual seawater temperature for central Chile in Las Cruces (Storch et al., 2009a). Females were fed using *L. spicata* collected on the coast of the ECIM.

#### Larval collections

After spawning, Zoea I larvae were collected using a net of 0.23-mm pore size mesh and used directly in the 48-h experiments. Collected larvae were selected based on physiological and behavioral features like color (black) and swimming activity (circular and non-midwater swimming). Twenty larvae were selected for each replicate of each experiment and placed in 400-mL aquaria.

#### Requirements of experimentation

**Fig. 1:**
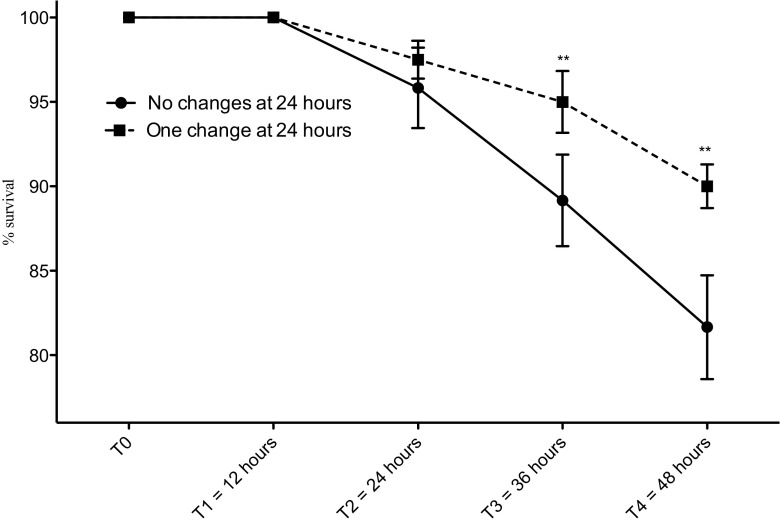
Requirements of experimentation: Survival of the Zoea I of *Taliepus dentatus* during the first 48 h after hatching with and without replacement of the seawater at 24 h. A two-way ANOVA (*p* < 0.01) completed by a Bonferroni posttest was performed and significant results are indicated (**)

The optimal growth conditions were preliminarily determined. A total of 120 larvae were used to realize the experiment by changing the medium after 24 h (20 larvae in 400-mL sea water filtered in triplicate) and without changing the medium (20 larvae in 400-mL sea water filtered in triplicate) (Fig. 1).

#### The effect of copper on Zoea I of *T. dentatus*

The experiment was conducted for 48 h in triplicate for each concentration with a total of 20 larvae by replicate in 400 mL, changing the seawater at 24 h and counting the mortalities each 12 h. Six treatments, consisting of filtered seawater with increasing copper concentrations, were included. Based on preliminary experiments, copper treatments included the nominal concentrations of 48, 68, 140, 200, 283, and 407 μg Cu L^−1^ as CuCl_2_ (Titrisol®, Merck, Germany). A treatment of seawater with no added copper was also included as control.

#### The effect of *L. spicata* exudates on toxicity of copper on Zoea I of *T. dentatus*

The experiment was conducted for 48 h in triplicate for each concentration with a total of 20 larvae by replicate in 400 mL, changing the seawater at 24 h and counting the mortalities each 12 h. Seven treatments, consisting of filtered seawater enriched in *L. spicata* exudates with increasing copper concentrations, were included. Based on preliminary experiments, copper treatments included the nominal concentrations of 100, 143, 204, 292, 416, 595, and 850 μg Cu L^−1^ as CuCl_2_ (Titrisol®, Merck, Germany). A treatment of seawater with no added copper was also included as control.

#### The effect of an exposure of ovigerous females of *T. dentatus* to an environmental concentration of copper on Zoea I of *T. dentatus*

Before spawning, selected mature females were conditioned at a nominal copper concentration of 25 μg Cu L^−1^ as CuCl_2_ (Titrisol®, Merck, Germany), corresponding to the mean measured in a contaminated area (Andrade et al. 2006). The experiment was conducted for 48 h in triplicate for each concentration with a total of 20 larvae by replicate in 400 mL, changing the medium at 24 h and counting the mortalities each 12 h. Six treatments consisting of filtered seawater with increasing copper concentrations were included. Based on preliminary experiments, copper treatments included the nominal concentrations of 48, 68, 140, 200, 283, and 407 μg Cu L^−1^ as CuCl_2_ (Titrisol®, Merck, Germany). A treatment of seawater with no added copper was also included as control.

### Statistical analysis

To determine the 48-h 50% effective concentration (EC_50_) for the algae spores and the 48-h lethal concentration for 50% of the population (LC_50_) for crab larvae, data were transformed into Log Cu, and then they were normalized. Non-linear regressions, with a maximum of 100 and a minimum of 0 effect percentage, were realized to determine the value of the 48-h EC_50_ and of the 48-h LC_50_, respectively. Statistical analyses were performed on Graphpad Prism 5™. The regressions undergo the following equation, Y = Low value + (100%-low value) / (1 + 10^ ^((LogEC50-X) *HillSlope)^), with X the logarithm of the concentration and Y the evaluated toxicological end point. Y starts with the lowest value and goes at 100% with a line in sigmoid function. This equation is identical to “four-parameter logistic equation.” A two-way ANOVA (*p* < 0.001) completed by a Bonferroni’s test was realized on Graphpad Prism 5™ after data normalization to evaluate difference between times of exposition for each treatment (copper; copper + exudates; exposition before hatching to copper) of *T. dentatus*.

## Results

### The effect of *L. spicata* exudates on toxicity of copper on spore germination of *L. spicata* and *U. lactuca*

The 48-h EC_50_ values for spore germination of *U. lactuca* and *L. spicata*, in response to different copper treatments with and without exudates of *L. spicata*, are shown in Table 1. The toxicity of metals varies with species and treatments. Spore germination of *U. lactuca* yielded different EC_50_ values (48-h EC_50_ = 8 μg Cu L^−1^) by comparison to *L. spicata* (48-h EC_50_ = 23 μg Cu L^−1^) in copper medium without exudates. The 48-h EC_50_ for spore germination in the presence of exudates of *L. spicata* evolved in the same way but were still different for each species (48-h EC_50_ for *U. lactuca* = 119 μg Cu L^−1^ and 48-h EC_50_ for *L. spicata* = 213 μg Cu L^−1^).

**Table 1:** Forty-eight-hour EC50 for spore germination of *Ulva lactuca* and *Lessonia spicata* with and without *L. spicata* exudates

Species	48-h EC50 without exudates	48-h EC50 with exudates
*Ulva lactuca*	8 μg Cu L^−1^	23 μg Cu L^−1^
*Lessonia spicata*	119 μg Cu L^−1^	213 μg Cu L^−1^

### The effect of copper on Zoea I of *T. dentatus*

**Fig. 2:**
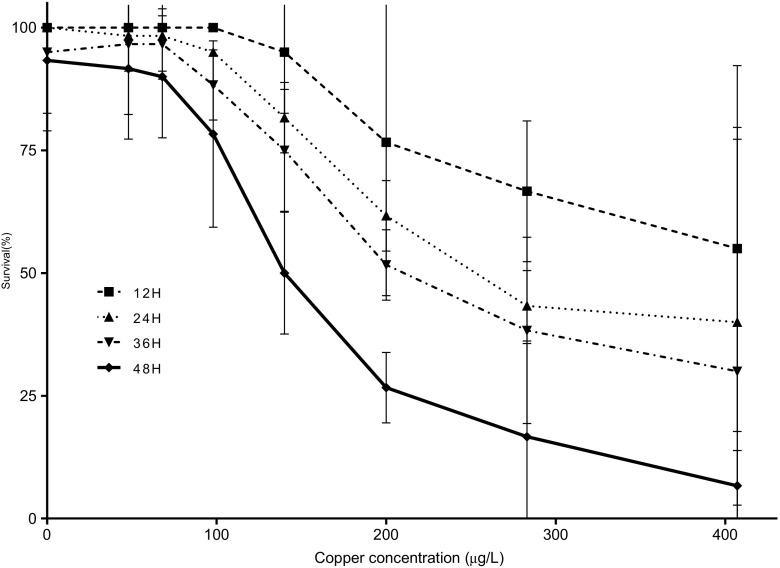
The effect of copper on Zoea I of *Taliepus dentatus*. Evolution of the mortality was registered every 12 h at all the used concentration of copper and tested by two-way ANOVA completed by a Bonferroni posttest

The tolerance of Zoea I larvae to copper varied with the concentrations of metal and the time of exposition (Fig. 2). Survivorship in the control and copper treatments up to 68 μg Cu L^−1^ was high (>91%). Survivorship declined progressively from 78% at 98 μg Cu L^−1^ to 7% at 407 μg Cu L^−1^ at 48 h. A similar copper dose-dependent survivorship occurred for each time point (12, 24, 36 h). The 48-h LC_50_ was 144 μg Cu L^−1^ (95% confidence intervals: 137 to 152 μg Cu L^−1^) (Table 2). From copper concentration equal to 100 μg Cu L^−1^ and higher, particular phenotypes were observed with body depigmentation, local hyperpigmentation of the telson, and hypopigmentation of the eyes (data not shown).

**Table 2:** Forty-eight-hour LC50 of Zoea I of *Taliepus dentatus* with and without *L. spicata* exudates and 48-h LC50 of Zoea I of ovigerous females of *T. dentatus* exposed to an environmental concentration of copper

Species	48-h LC50 without exudates	48-h LC50 with exudates	48-h LC50 with exposition to copper before hatching
*Taliepus dentatus*	144 μg Cu L^−1^	249 μg Cu L^−1^	166 μg Cu L^−1^

### The effect of *L. spicata* exudates on toxicity of copper on Zoea I of *T. dentatus*

**Fig. 3:**
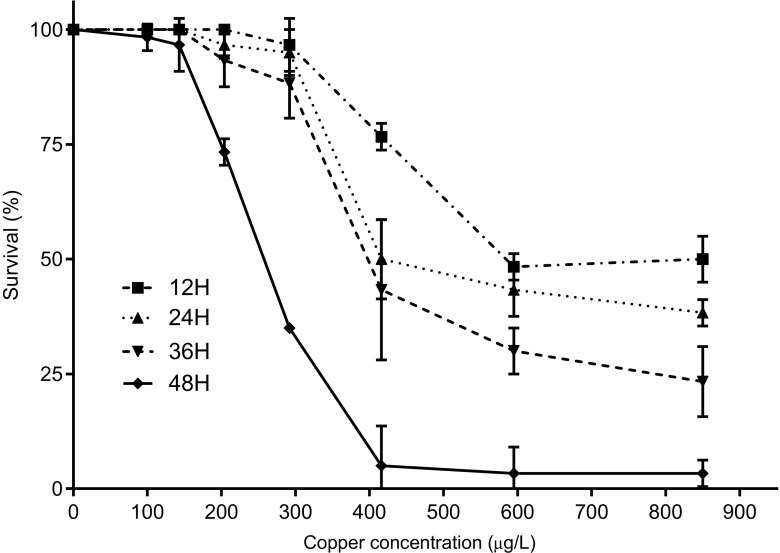
The effect of *Lessonia spicata* exudates on toxicity of copper on Zoea I of *Taliepus dentatus.* Evolution of the mortality was registered every 12 h at all the used concentration of copper and tested by two-way ANOVA completed by a Bonferroni posttest

The tolerance of Zoea I larvae to copper with *L. spicata* exudates varied with the concentrations of metal and the time of exposure (Fig. 3). Survivorship in the control and copper treatments up to 143 μg Cu L^−1^ was high (>98%). Survivorship declined progressively from 73% at 204 μg Cu L^−1^ to 3% at 595 μg and 850 μg Cu L^−1^ at 48 h. The 48-h LC_50_ was 249 μg Cu L^−1^ (95% confidence intervals 241 to 258 μg Cu L^−1^) (Table 2). From copper concentration equal to 143 μg Cu L^−1^ and higher, particular phenotypes were observed with body depigmentation, local hyperpigmentation of the telson, and hypopigmentation of the eyes (data not shown).

### The effect of an exposure of ovigerous females of *T. dentatus* to an environmental concentration of copper on Zoea I of *T. dentatus*

**Fig. 4:**
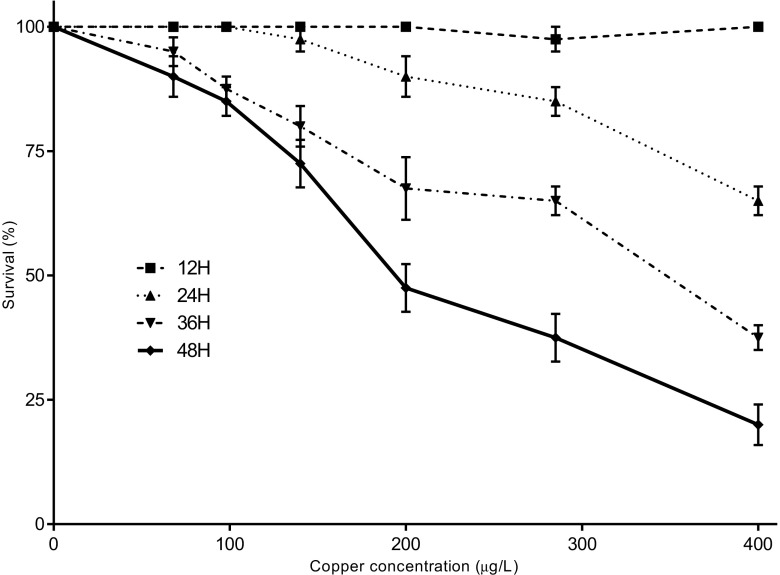
The effect of an exposure of ovigerous females of *Taliepus dentatus t*o an environmental concentration of copper on Zoea I of *T. dentatus*. Evolution of the mortality was registered every 12 h at all the used concentration of copper and tested by two-way ANOVA completed by a Bonferroni posttest

The tolerance of Zoea I larvae, previously exposed to copper before hatching, varied with the concentrations of metal and the time of exposition (Fig. 4). Survivorship in the control and copper treatments up to 68 μg Cu L^−1^ was high (>95%). Survivorship declined progressively from 85% at 98 μg Cu L^−1^ to 20% at 400 μg Cu L^−1^ at 48 h. The 48-h LC_50_ was 166 μg Cu L^−1^ (95% confidence intervals 151.7 to 181.1 μg Cu L^−1^) (Table 2). No apparent delay in hatching or loss of eggs was observed after exposition of ovigerous females to a copper concentration of 25 μg Cu L^−1^.

## Discussion

The results demonstrate that (i) the brown kelp *L. spicata* has the capacity to produce exudates that are able to protect algae and invertebrates associated with the kelp ecosystem. Certainly, the presence of exudates has reduced, in all cases, the toxicity of copper in early developmental stages of life cycles of seaweeds, such as spores of *U. lactuca* (Chlorophyceae) and *L. spicata* (Phaeophyceae) and in larvae Zoea I of the kelp crab *T. dentatus* (Crustacea)*.* The ecotoxicological assays (ii) on the crab have demonstrated that larval stage might have the tolerance and the capacity to survive in a copper-enriched ecosystem. Finally, it is possible to consider the fact that (iii) a non-lethal exposure of ovigerous females before hatching may enhance the tolerance of larvae to copper stress.

### The effect of *L. spicata* exudates on toxicity of copper on *L. spicata* and *U. lactuca* spore germination

Pollutants, such as metals, decreased spore liberation, mobility, and spore germination in different algae (Agrawal 2009). Copper toxicity varies with species as demonstrated by the present study on *U. lactuca* and *L. spicata* as well in other seaweeds (review in Han et al. 2011). Obtained results for spore germination showed different sensitivities for 48-h EC_50_ with 8 μg Cu L^−1^ for *U. lactuca* and 23 μg Cu L^−1^ for *L. spicata*. In this sense, spores of *U. lactuca* are much more sensitive to copper than the to kelp. However, comparatively to other brown seaweeds, *L. spicata* is less tolerant. Indeed, the brown kelps *Ecklonia radiata* and *Saccharina japonica* (formerly *Laminaria saccharina*) and the brown algae *Hormosira banksii* are more tolerant with EC_50_ values for spore germination are ranged from 320 to 470 μg Cu L^−1^ (48-h EC_50_) (Bidwell et al. 1998), >500 μg Cu L^−1^ (no effect, 12-h EC_50_) (Han et al. 2011), and 170 μg Cu L^−1^ (48-h EC_50_) (Myers et al. 2006), respectively.

Furthermore, as previously demonstrated (Andrade et al. 2010), *L. spicata* has the capacity to produce copper complexing exudates. To go further in their characterization, it can be hypothesized that these exudates might give protection for early stages of seaweeds, which develop under their canopy. In this study, it was demonstrated that exudates decrease copper toxicity on spores of *U. lactuca* and *L. spicata* up to 9 and 15 times (48-h EC_50_ values of 119 and 213 μg Cu L^−1^), respectively. These results are in line with previous studies as shown by a reduced copper toxicity on germlings of *Fucus vesiculosus* in the presence of organic dissolved carbon (Brooks et al. 2008), which might also affect the metal accumulation. However, metal toxicity and accumulation are not only influenced by the free metal ions but also by the exposure time as shown with the unicellular algae *Pseudokirchneriella subacapitata* exposed to cadmium (Paquet et al. 2015). Moreover, these defense mechanisms, even if *L. spicata* shows a great capacity to produce high quantity exudates, which able complexing copper for at least 48 h in a constant way (conditional stability constant (log K′) = 8.6 ± 0.3) (Andrade et al. 2010), the ability for kelp to face a permanent contamination by copper should be explored.

The data revealed that spores of both considered species, in the presence of kelps exudates, might germinate and continue with its development in an environment with moderately high levels of dissolved copper. However, previous experiments have shown that, even at very low concentrations (7.9 μg Cu L^−1^), without exudates, copper has deleterious effects on *L. spicata*. It affects the spore release, totally interrupting the early development of the spore after they settle, which prevents the growth of the macroscopic sporophyte phase (Contreras et al. 2007).

In addition, these results might be in line with what was observed with the experimental transplants of *L. berteorana* (formerly known as *L. nigrescens* until 2011/2012) in the environment of Chañaral (Correa et al. 2006), but recent molecular studies (Tellier et al. 2011; González et al. 2012) have divided the species in *L. spicata* (29°S–42°S) and *L. berteorana* (16°S–30°S). In this case, it has been demonstrated that juveniles were able to grow to mature and become reproductive (presence of sorus) with the capacity to release viable spores without completing their life cycle, even after several years of the survey (Correa et al. 2006; Contreras et al. 2007). Consequently, persistent copper levels in the seawater, even at low concentration, might be limiting the natural development of the kelp by an increase of lipoperoxide accumulation, which coupled with a low activity of antioxidant enzymes may lead to oxidative damage (Contreras et al. 2009a). Furthermore, presence of numerous invertebrates in a polluted environment might contribute to a local increase of herbivory (Correa et al. 1999) with a significant impact on the algae. In this sense, it has been demonstrated that grazers as the large chiton *Enoplochiton niger* are strong modifier of the spatial structure of the kelp-dominated low intertidal community in northern and central Chile (Aguilera et al. 2015).

With the apparently poor capacity for dispersal of the species (Faugeron et al. 2005), EC_50_ values obtained here show evidences that might explain why *U. lactuca* and *L. berteroana* are not naturally coming back in the Chañaral ecosystem. This fact is supported by the subsequent emergence of resistant species such as *Scytosiphon lomentaria* (Phaeophyceae) (Contreras et al. 2009b), *Ectocarpus siliculosus* (Phaeophyceae) (Ritter et al. 2014), and *Dyctiota kunthii* (Phaeophyceae) (Sordet et al. 2014). The adaptation capacities of *L. spicata*, the growth of spores together with the ability to reach the gametophytic phase in the presence of exudates, exposed at a permanent environmental concentration of copper, should be explored. To get more insights about the characteristics of the Chañaral ecosystem, the response to copper of early stages of *L. berteorana* must be further studied, as well as the capacity for these species to produce exudates complexing the metal.

### The effect of copper on Zoea I of *T. dentatus*

As demonstrated in numerous species, such as the fish model *Oryzias melastigma*, early stages are more sensitive to metals than are juveniles and adults (Guo et al. 2016). In crustacea, Zoea I remain the most sensible to elevated copper levels (Qiu et al. 2005). In fact, larvae are completely permeable to metals, unlike older stages or adults where the accumulation is occurring almost just in the gills, on the shell, or vía food uptake (Rainbow 1997). Based on that, the kelp crab *T. dentatus* is more tolerant to copper compared to the algae studied here, as illustrated by the 48-h LC_50_ of 144 μg Cu L^−1^. This result is close to what it is already known in other crustacea larvae such as the *Balanus amphitrite* (48-h LC_50_ of 145 μg Cu L^−1^) (Qiu et al. 2005). As it is observed previously in algae or amphibian (Leduc et al. 2015), copper tolerance is also species-specific among crabs (96-h LC_50_ of 219 μg Cu L^−1^ for *Chasmagnathus granulata* (Ferrer et al. 2006) and 48-h LC_50_ of 80 μg Cu L^−1^ for *C. granulata* (Ramachandran et al. 1997).

Furthermore, from copper concentration equal to 100 μg Cu L^−1^ and higher, particular phenotypes were observed with body depigmentation, local hyperpigmentation of the telson, and hypopigmentation of the eyes (data not shown). These results are in line with what is generally observed in crustacea. Indeed, copper has several impacts on development, such as increase of mortality and changes in the color of the chest and the eyes of the larvae (Lopez Greco et al. 2002; Lavolpe et al. 2004; Lahman et al. 2015). The present results were focused on the range of tolerance to copper of the Zoea I. However, to go further in the interactions between copper and larvae, we should extend our efforts to work with the other developmental stages because copper might inhibit the growth and delay on metamorphosis (Sanders et al. 1991; Wang et al. 2016).

Finally, no significant effects were observed to a copper concentration of 48 μg Cu L^−1^. Taking into account that copper concentrations close to the point of discharges in the copper impacted ecosystem of Northern Chile ranged between 8.8 and 34 μg Cu L^−1^ (Medina et al. 2005), it is possible to assume that copper might be not the limiting factor for *T. dentatus* as an herbivorous species (Pardo et al. 2007) with a presence in the contaminated area, which seems to be restricted to the places where *L. berteorana* is present (Medina et al. 2005), as a food source or a possible protection.

### The effect of *L. spicata* exudates on toxicity of copper on Zoea I of *T. dentatus*

Brown kelp *L. spicata* plays a protective role on larvae of *T. dentatus*. An increase of 48-h LC_50_ from 144 to 249 μg Cu L^−1^ is showing the presence of kelps exudates in the medium. These results are in line with other studies about the protective effect of exudates on larvae as with the polychaete *Hydroides elegans* (Wong et al. 2006) or with the freshwater mussel *Lampsilis siliquoidea.* This mussel is a very sensitive species (Jorge et al. 2013) where dissolved organic matter present in a sufficient amount in the environment has a protective effect against copper (Jorge et al. 2013). Very little is known about the protection of organic ligands for crustacea larvae against metals with some cases where exudates do not have an efficient protective role. For example, the life span of *Daphnia magna* is more influenced by food availability than by interactions between metals and algal exudates in case of chromium contamination (Gorbi et al. 2002). To go further in the interactions between copper and larvae, other developmental stages of the *T. dentatus* should be considered. The presence of these crabs in the contaminated area is restricted to the places where *L. berteorana* is present (Medina et al. 2005). This, combined with the implementation of a coastal restoration program (Correa et al. 2006), might possibly allow an extension of their repartition, protecting early stages of algae and invertebrates and decreasing the bioavailability of copper in parallel. However, the return of invertebrates in the contaminated ecosystem of Chañaral Bay, suppose that organisms living there will be exposed continually to copper pollution even if it is not lethal. This exposure could induce some modifications in organisms, leading potentially to acclimation and local microevolution (Medina et al. 2007; Pease et al. 2010) or their extinction.

### The effect of an exposure of ovigerous females of *T. dentatus* to an environmental concentration of copper on Zoea I of *T. dentatus*

Preliminary field study on newly established populations in the contaminated ecosystem of Chañaral Bay showed that ovigerous females are accompanied by some apparent differences in size and sex proportions (data not shown), which are also in line with other studies (Chen et al. 2005; Agra et al. 2011). It means that crabs are permanently exposed to copper concentrations, ranging between 8.8 and 34 μg Cu L^−1^ (Medina et al. 2005). In this context, when the exposed ovigerous females to an environmental concentration of copper of 25 μg Cu L^−1^, no apparent delay in hatching and no loss of eggs were observed. According to the literature, this is not specific to the considered species. In the horseshoe crab *Limulus polyphemus*, a high percentage of embryos that survive and hatch is generally unaffected by metal exposure, except at the highest copper concentration, even if there were significant delay in developmental rate (Hamilton et al. 2015).

In this study, Zoea I presented, after 48 h, an apparent better resistance to copper compared to the initial calculation, with a new 48-h LC_50_, which is about 166 against 144 μg Cu L^−1^. These results give evidence for the general hypothesis that preexposition could enhance the capacity to resist to a stressor like copper. Thus, metal acclimation can be defined as enhanced tolerance to abnormally lethal metal concentration as a result of exposure to a sublethal level (Wang et al. 2016). They are in line with other studies, such as in the porcelain crab, *Petrolisthes galathinus* (Lopez Greco et al. 2002), or the amphipod *Gammarus aequicauda* (Prato et al. 2013), where larvae exposed to copper during their embryonic development are more tolerant to contamination. Local acclimations are also observed in some other invertebrates (Pease et al. 2010). However, increase of tolerance to copper should be also examined during the entire life cycle of *T. dentatus*. Indeed, the effect of a constant exposure can be negative, as observed with the grass shrimps *Paleomonetes pugio* exposed continuously to copper, which exhibit an inability to produce viable embryos, precluding completion of the lifecycle, despite no lethal effects on larva, juvenile, or adult life stages. These might be due to a possible relocation of the energy for reproduction in the antioxidant defenses (Manyin and Rowe 2010).

Finally, even if the tolerance is good, copper might induce changes such as epigenetic modifications, genetic mutations, behavioral changes, or perturbations of chemoreceptors on larvae and/or adult stages as observed in the crayfish *Orconectes rusticus*, where copper, before it is lethal, alters the behavioral capacities for orientations and localizations of food sources (olfaction) (Bini and Chelazzi 2006; Lahman and Moore 2015). Furthermore, modifications of the aggressor or the defense against aggressor in the hermit crab *Pagurus bernhardus* have also been shown (White et al. 2013) and the locomotor activity of the *G. fossarum* is proved to be affected (Arce Funck et al. 2013). At the molecular level, some aspect as epigenetic modifications must also be considered (Burggren 2014). For example, mechanisms such as DNA methylation are crucial during the development of different marine organisms (Riviere et al. 2013) and the influence of the environment, even copper (Marshall 2008), has been clearly demonstrated in some cases (Fellous et al. 2015) with a significant transgenerational impact (Marshall 2008; Thor and Dupont 2015).

## Concluding comments

The present study demonstrates for the first time that the brown kelp *L. spicata*, a key species in the coastal environment of the Southeast Pacific coast, has the capacity to produce exudates with a protective role regarding copper contamination on different species of algae and invertebrates associated with the kelp ecosystem. Additionally, Zoea I of *T. dentatus* might be able to develop and to acclimate in the contaminated ecosystem of Chañaral Bay, and copper may not be directly the limiting factor for the crab, but for the kelp could be. To get more insight in this hypothesis, it must be studied the effect of a permanent exposure to copper at polluted environmental concentrations, along the complete lifecycle of the *T. dentatus*, in order to appreciate if the metamorphosis is done and if the adults are able to produce viable embryos.

Field experiments should also lead to the study of the crab at a population level with the idea that some acclimations might be present in the impacted area, as suggested by the preliminary results. On the other hand, responses to copper of *L. berteorana* must be studied to elucidate exactly why the kelp is not coming back yet naturally. The capacity for spores to germ and develop, which are coming from individuals that have grown under permanent copper exposure, has to be known, as well as the potential acclimation capacity for the species and its ability to produce exudates complexing copper. Thus, in this context of small populations (meaning potentially low genetic diversity) and transgenerational contaminations, molecular studies should focus on the characterization of epigenetic mechanisms as a potential for long-term adaptation of crustacean and algae. Knowing these mechanisms, it could be the next generation of biomonitoring tools in marine organisms.
